# Components of the Lectin Pathway of Complement in Solid Tumour Cancers

**DOI:** 10.3390/cancers14061543

**Published:** 2022-03-17

**Authors:** Maciej Cedzyński, Anna S. Świerzko

**Affiliations:** Laboratory of Immunobiology of Infections, Institute of Medical Biology, Polish Academy of Sciences, Lodowa 106, 93-232 Łódź, Poland; aswierzko@cbm.pan.pl

**Keywords:** cancer, collectin, complement, ficolin, lectin pathway, MBL-associated serine protease (MASP)

## Abstract

**Simple Summary:**

Cancer is a major cause of human deaths. The complement system is an important branch of the innate immune response that can be activated via three distinct pathways (classical, alternative, lectin). Complement activation may contribute to cancer prevention, but on the other hand, its over- or chronic activation can be harmful for the host. In this short review, we discuss the dual role of the lectin pathway of complement activation in human solid cancers, including those of the female reproductive system, lung, and alimentary tract, with emphasis on its cross-talk with other enzyme-dependent cascades.

**Abstract:**

The complement system is an important branch of the humoral innate immune response that can be activated via three distinct pathways (classical, alternative, lectin), contributing to keeping/restoring homeostasis. It can also interact with cellular innate immunity and with components of acquired immunity. Cross-talk between the complement system and other enzyme-dependent cascades makes it a more influential defence system, but on the other hand, over- or chronic activation can be harmful. This short review is focused on the dual role of the lectin pathway of complement activation in human solid tumour cancers, including those of the female reproductive system, lung, and alimentary tract, with emphasis on the aforementioned cross-talk.

## 1. Introduction

The complement system has been studied since the late 19th century, but some aspects of its involvement in human/animal health and disease are still unclear. Although it is commonly considered as a part of the immune response that can be activated via three distinct pathways (classical, alternative, lectin, all leading to the terminal or common pathway), contributing to keeping/restoring homeostasis, it appears in fact much more complex and multifaceted than expected previously.

The classical pathway (CP) is initiated upon binding of the C1qr_2_s_2_ complex (where C1q is an oligomeric pattern-recognizing molecule (PRM), while C1r and C1s are zymogens) to the complex of antigen and antibody (of IgG or IgM class). C1r is then auto-activated and in turn activates C1s. Its substrates are C4 and C2, enabling formation of an active C3 convertase (C4b2a complex). That cleaves the C3 molecule, resulting in release of C3a (anaphylatoxin) and C3b, which binds to C4b2a, thus forming the C4b2a3b complex (C5 convertase). The CP may also be activated independently of immunoglobulins, by C-reactive protein (CRP), β-amyloid, serum amyloid P (SAP), bacterial lipopolysaccharides (LPS) and nucleic acids, apoptotic or necrotic cells, and viral proteins [1,2]. The alternative pathway (AP) is activated constantly by the formation of C3 convertase, resulting from spontaneous hydrolysis of C3, and therefore has to be controlled efficiently by soluble and membrane-bound inhibitors. The product of C3 hydrolysis is C3(H_2_O), corresponding functionally to C3b. That binds to factor B, which then undergoes cleavage by factor D, which results in release of the Ba fragment and formation of C3 convertase, C3(H_2_O)Bb. Its half-life is short, but upon binding to the surface of pathogens (or released components of their cell-envelopes such as LPS), virus-infected/damaged self-cells, CRP, protein A, or cobra venom factor (CVF), and stabilization by properdin (factor P) and Mg^2+^, it is prolonged 10-fold. Such stabilization enables amplification of AP by producing more C3b fragments, and consequently, C3bBb complex (AP C3 convertase). After the binding of another C3b to the C3bBb complex, the AP-specific C5 convertase (C3bBb3b) is formed [1,2].

The lectin pathway (LP) is initiated upon binding of mannose-binding lectin (MBL), collectin-10 (CL-10), collectin-11 (CL-11), or a ficolin (ficolin-1, -2, -3), complexed with proteins of the MASP family (MBL-associated serine proteases (MASP-1, -2, -3) and related non-enzymatic proteins (MAp19 and MAp44)) to microbial polysaccharides/glycoconjugates or altered (apoptotic/necrotic/cancerous) autologous cells, with abnormal glycosylation pattern. Collectins and to some extent also ficolins, structurally resemble C1q, while MASP-1 and MASP-2 are homologues of C1r and C1s, respectively. Therefore, after binding of the collectin/ficolin–MASP complex to the target structure, auto-activated MASP-1 activates MASP-2, which in turn cleaves C4 and C2, thus enabling formation of C4b2a (C3 convertase). Further events proceed following CP activation [1,2].

The CP/LP or AP C5 convertase initiates the terminal pathway by digestion of C5 to C5a (anaphylatoxin which is released) and C5b (bound to the target) fragments. Next, C6, C7, C8, and 10–15 C9 molecules associate, forming a transmembrane pore, leading to cell lysis [1,2].

Soluble C1-esterase inhibitor (C1INH) and C4b-binding protein (C4bp) regulate activation of classical and lectin pathways by inhibiting the formation of C3 and C5 convertases formation, respectively. The fluid phase AP regulators, factors H and I, inhibit complement activation at the same stages. Furthermore, autologous cells are prevented from complement-dependent lysis by such membrane-bound molecules as CD35, CD46, CD55, and CD59 [1,2]. The overexpression of complement regulators or down-regulation of common pathway factors are major mechanisms of evasion of the host immune response by tumour cells [3]. The cross-talk among activation pathways and with other enzyme-dependent cascades make it a more comprehensive defence system, but on the other hand, over- or chronic activation can contribute to severe adverse effects. In the context of cancer, complement activation may lead to direct lysis of altered cells or contribute to their elimination via complement-dependent cellular cytotoxicity (CDCC). Moreover, it may enhance the effect of antibodies, including therapeutic mAb. However, it may also promote neoplastic transformation, cell proliferation, tumour growth, epithelial–mesenchymal transformation (EMT), angiogenesis, and metastasis [4,5,6,7]. The beneficial or harmful effects for the host depend on the sensitivity of cancer cells to complement attack, sites of its activation, and the composition of the tumour microenvironment [3]. Roumenina et al. [3] and Revel et al. [7] provided in-depth analysis of multifaceted associations of complement with malignancy. They proposed classification of solid tumour cancers into four groups: (i) cancers in which generally high expression of complement genes predicts better prognosis and therefore complement plays a protective role (mesothelioma, prostate adenocarcinoma, sarcoma, skin cutaneous melanoma); (ii) cancers where high C3 expression only acts protectively (adrenocortical carcinoma, kidney chromophobe carcinoma, thyroid carcinoma); (iii) cancers in which high expression of complement genes is associated with poor prognosis (colon adenocarcinoma, oesophageal carcinoma, glioblastoma, kidney clear cell carcinoma, lower grade glioma, lung squamous carcinoma, rectum adenocarcinoma, stomach adenocarcinoma, uterine corpus endometrial carcinoma, uterine carcinosarcoma, uveal melanoma); (iv) cancers where the role of complement is uncertain (bladder carcinoma, cervical squamous carcinoma, cholangiocarcinoma, diffuse large B cell lymphoma, head and neck squamous cell carcinoma, invasive breast carcinoma, kidney papillary cell carcinoma, lung adenocarcinoma, ovarian serous cystadenocarcinoma, pancreatic adenocarcinoma, thymoma). Changes of expression of complement-associated genes may occur both locally (in tumour and its microenvironment) and systemically [3,7]. Although not all those associations were statistically significant, such a classification may be helpful in looking for new prognostic markers or future therapeutic procedures.

**Figure 1:**
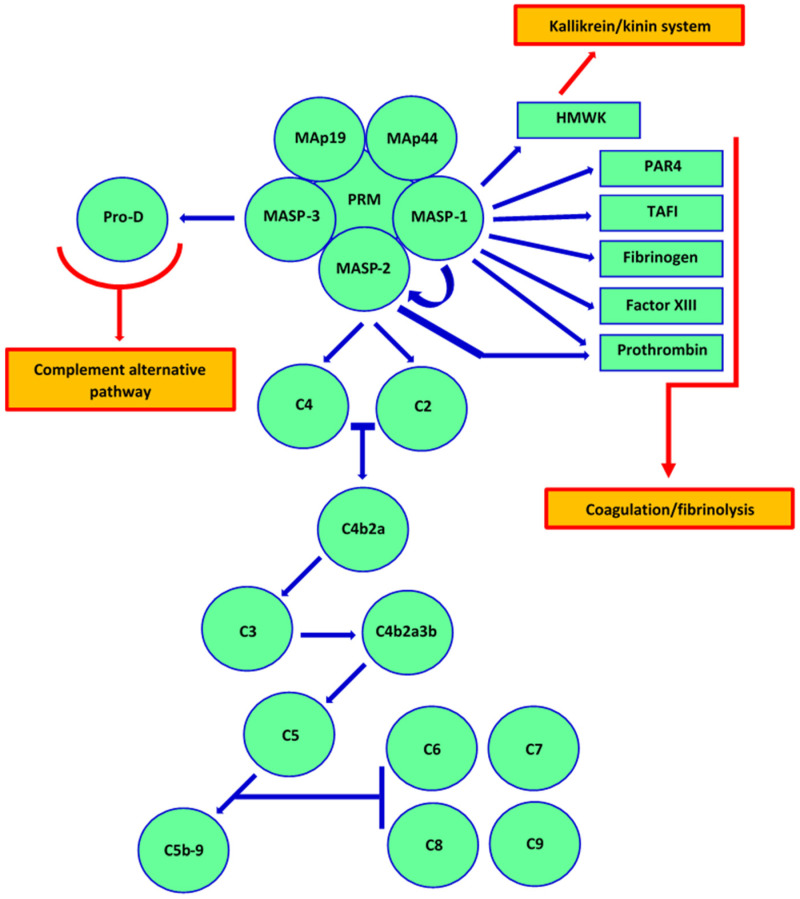
Activation of complement via the lectin pathway and its cross-talk with other processes. MASP-1 participates in the coagulation/fibrinolysis cascade via activation of prothrombin and factor XIII, and cleavage of thrombin-activatable fibrinolysis inhibitor (TAFI). It furthermore interacts with protease-activated receptor 4 (PAR4). MASP-1 cleaves high molecular weight kininogen as well, resulting in release of bradykinin, thus cross-talking with the kallikrein–kinin system. MASP-2 activates prothrombin (coagulation pathway), while MASP-3 activates pro-factor D (cross-talk with alternative pathway of complement). As mentioned within the main text, LP shares C3 and C5 convertases with the classical pathway. PRM—pattern-recognizing molecule (collectin or ficolin); HMWK—high molecular weight kininogen.

In our recent review [8], we discussed in detail factors specific for the lectin pathway (LP) and their associations with haematological malignancies. Here, we would like to shed some light on the dual role of the LP in human solid tumour cancers. It has to be remembered that the LP extensively cross-talk not only with the classical (the same C3/C5 convertases) and alternative (via MBL-associated serine protease-3 (MASP-3), activating pro-factor D) pathways of complement [9,10,11] but also with the coagulation/fibrinolysis and the kallikrein/kinin systems [12,13,14,15,16,17,18,19] (Figure 1). Especially, MASP-1 appears to be a pluripotent enzyme, essential not only for complement activation. It also contributes to the coagulation process via activation of prothrombin and factor XIII as well as to protection from clot lysis from cleavage of thrombin-activatable fibrinolysis inhibitor (TAFI) [13,17,20]. It furthermore interacts with protease-activated receptor 4 (PAR4), cathepsin B, trypsin, gingipain R, and kallikrein 14 [21,22,23,24]. The last mentioned is involved not only in activation and aggregation of platelets but affects survival, proliferation, and migration of cancer cells. MASP-1 cleaves high molecular weight kininogen as well, resulting in release of bradykinin [15,22]. MASP-2 in turn, activates complement C4 and C2 and also prothrombin. It is able to cleave kininogen but with no creation of bradykinin [12,15].

## 2. Gynaecological Cancers

The associations of lectin pathway factors with gynaecological cancers are multifaceted and often unclear. Numerous reports demonstrated that their low expression impaired activity or related genetic variants favour carcinogenesis. The O/O and A/O *MBL2* genotypes (where O corresponds to any of the variant alleles commonly called D, B, and C, localized to exons 52, 54, and 57 of the first exon, respectively, while A refers to the wild type) were found to be associated with ovarian cancer [25,26,27]. Furthermore, the ovarian expression (at both mRNA and protein level) of *FCN2* and *FCN3* (ficolin-2 and ficolin-3, respectively) were lower in tissue sections from malignant tumours compared with benign tumours or normal ovaries [28]. Similarly, expression of the *MASP1* (MASP-1, MASP-3, MAp-44, the latter lacking enzymatic activity) gene was reported to be higher in normal endometrium tissue, compared with samples from patients diagnosed with endometriosis or endometriosis-associated ovarian cancer. That was, however, in contrast with other genes involved in complement activation (*C3*, *C4A*, *CFH*, *CFD*, and *CFB*) [29]. Recently, Jang et al. [30] observed lower expression of the *FCN3* gene in tissue samples from invasive carcinoma of the breast (BRCA), compared with normal tissue.

The beneficial effect of LP activation (or unfavourable result of its low level) in relation to cancer may also reflect its antimicrobial potency. It is well known that certain infections may induce or promote carcinogenesis. The risk of ovarian cancer, for example, was found to increase after infections with certain pathogens, including viruses (cytomegalovirus, CMV; Epstein–Barr virus, EBV; human papillomavirus, HPV; human immunodeficiency virus, HIV), bacteria (*Neisseria gonorrhoeae, Chlamydia trachomatis*) or parasites (reviewed by Sipos et al. [31]). However, the role of dysbiosis and commensal microorganisms are gaining attention, especially in gynaecological malignancies. In another recent review, Alizadehmohajer et al. [32] profoundly discussed several mechanisms mediated by the microbiome, including stimulation of proliferation or cell death, dysregulation of the immune response, induction of metabolic changes, and DNA damage. Interestingly, some bacterial products have hormone-like properties that may favour development of gynaecological or pancreatic cancers (reviewed by Sipos et al. [31] and Borella et al. [33]). It is considered that various types of cancer are associated with specific microbial patterns. Banerjee et al. [34] identified bacteria from the genera *Abiotrophia*, *Bacillus*, *Enterococcus*, *Erysipelotrix*, *Geobacillus*, *Lactobacillus*, *Lactococcus, Listeria*, *Pediococcus*, *Peptoniphilus*, and *Streptococcus* to form a community (called oncobiome) related to ovarian cancer [32,34]. Generally, OC was reported to be characterised by dysbiosis (oncobiosis) affecting vagina, upper genital tract, alimentary tract, peritoneum, blood, and ovary/tumour itself (reviewed by Sipos et al. [31]). The susceptibility to infection, its course, and outcome depend on a variety of factors, including the interplay between invading pathogens, the host’s microbiome, and the innate/acquired immune responses. Opsonization with lectin pathway-associated pattern-recognizing molecules followed by phagocytosis or complement activation is one of several mechanisms leading to elimination of certain microbial carcinogens. Wang et al. [35], from a meta-analysis of published data, concluded that Caucasian women carrying *MBL2* O alleles are at higher risk of HPV infection and subsequent development of cervical cancer.

On the other hand, high expression of LP-associated genes or related complement overactivation may be associated with promotion of carcinogenesis, faster disease progress, and/or worse prognosis. Although, as mentioned, low MBL-conferring genotypes are risk factors for development of OC, in women with malignant ovarian tumours carrying A/A genotypes, median MBL levels (as well as MBL–MASP-2 complex activities) were higher compared with A/A homozygotes found in reference groups (healthy women and patients diagnosed with benign tumours) and correlated with C-reactive protein (CRP) [25,26]. Moreover, primary MBL deficiency (defined as carrying O/O or LXA/O genotype, where LX is related to promoter SNP, at positions -550 (H/L) and -221 (Y/X)) was associated with longer survival after cytoreduction in OC patients [26]. That finding was supported by data published by Siamakpour-Reihani [36], who found high expression of several immune response-associated genes (including *MBL2*) to predict shorter survival in patients suffering from high-grade ovarian serous carcinoma. Furthermore, in contrast to ficolin genes, the ovarian expression of *MBL2* and *MASP2* was significantly higher in ovarian sections from malignant tumours compared with benign tumours or normal tissue [25,26,28]. Recently, Sahar et al. [37] found the presence of *MBL2* gene-specific mRNA in leiomyoma tissue but not in normal myometrium. They reported also higher expression of other LP-related genes (*MASP2*, encoding for MASP-2 and non-enzymatic MAp-19) in diseased samples [37]. MASP-2 concentrations were in turn significantly higher in sera from patients suffering from uterine corpus endometrial carcinoma (UCEC) compared with controls [38]. The MASP-1 expression was moreover found up-regulated (in a stage-dependent manner) in women diagnosed with uterine cervical cancer [39]. Maestri et al. [40] reported higher concentrations of MASP-1, MASP-2, and MAp-19 in sera from patients suffering from invasive cancer, in comparison with those diagnosed with cervical intraepithelial neoplasia (CIN)-I–III.

Regarding potentially harmful effects of ficolins, Szala et al. [28] found higher median serum concentrations of ficolin-3 and ficolin-2 from OC patients than in the aforementioned controls [26]. Earlier, ficolin-3 was considered a potential disease marker [41]. Moreover, the *FCN2* gene overexpression was related to leiomyoma [37] as well as uterine cervical cancer, at both mRNA [42] and protein [43] levels.

These data suggest that the role of lectin pathway factors in gynaecological cancers depends on disease type, its stage, and its site. For example, low activity of the MBL–MASP complex seems to favour development of ovarian cancer [25,26,27]. However, its high activity may contribute to disease progression, which was thought to be related to complement activation induced by recognition of agalactosylated/sialylated IgG glycoforms by the lectin [44]. High local expression of both *MBL2* and *MASP2* genes was also associated with malignant tissue [26]. In contrast, as mentioned, MBL deficiency was found associated with longer survival of patients [26]. The picture seems even more complicated when the involvement of LP in inflammatory processes (beneficial when well controlled but adverse when leading to hyper- or chronic activation), coagulation/fibrinolysis, and microbiome composition is considered.

## 3. Lung Cancer

Lung cancer (bronchial/bronchogenic carcinoma) is a common group of malignancies in both men and women and is a leading cause of cancer-related mortality with a 5-year survival rate of only 15%. There are four main types of lung cancer: adenocarcinoma, squamous cell carcinoma, large cell carcinoma (collectively known as non-small cell cancers), and small cell carcinoma. Again, some published reports suggest a protective effect of high expression/activity of LP-specific factors, while the others demonstrate the opposite associations. Promising data were published by Jang et al. [30] who found the expression of the *FCN3* gene to be lower in lung adenocarcinoma (LUAD) than in normal tissue. Similar phenomena were noted in lung squamous cell carcinoma (LUSC) and several cancers of other organs (mentioned elsewhere in this review). Interestingly, the ficolin-3 expression in LUAD patients was found to correlate inversely with mortality and was therefore supposed to be useful as a prognostic factor of life expectancy. Furthermore, based on results from cell lines, Jang et al. [30] suggested that ectopically-expressed *FCN3* is a tumour suppressor gene, acting by induction of cell cycle arrest and apoptosis via endoplasmic reticulum stress. Another potential diagnostic/prognostic marker for lung adenocarcinoma may be collectin-11 in serum and urine. Its concentrations were found to be lower in metastatic disease than from patients with primary tumours. It was suggested that the collectin-11 serum/urine ratio may be an indicator of disease progression and to be useful for determination of its stage [45].

Kang et al. [46] observed marked overexpression of the *MASP1* gene in lung squamous cell carcinoma (in contrast to adenocarcinoma) and suggested it was an oncogene that could be targeted. Later, Chen et al. [47] reported significantly higher concentrations of MASP-2 in sera from patients diagnosed with non-small cell lung cancers compared with those with benign tumours or healthy controls; they suggested MASP-2 (in combination with isocitrate dehydrogenase 1 (IDH1) and the cytokeratin 19 fragment, Cyfra21-1) could serve as a disease marker.

Therefore, again it seems that both impaired and excessive activity of the lectin pathway may be involved in carcinogenesis and/or disease progression, the first via insufficient elimination of altered-self cells, and the second via disruption of homeostasis associated with boosted/chronic inflammatory processes.

## 4. Alimentary Tract Cancers

In this section, we review the literature concerning lectin pathway components in cancers of the oesophagus, stomach, pancreas, bowel, and liver. Regarding associations of low expression/activity of lectin pathway factors with alimentary system malignancies, Wang et al. [48] found the *MBL2* codon 54 B variant to be over-represented in younger (aged ≤ 65 years) Japanese patients diagnosed with gastric cancer. Other authors suggested the significance of the D (codon 52) allele in Europeans [49,50]. Significantly lower MBL concentrations were found in sera from patients suffering from hepatocellular carcinoma (HCC) (both HBs-positive and -negative), compared with controls [51]. Presently, growing attention is being paid to epigenetic mechanisms of regulation of gene expression, including involvement of microRNA (miRNA) in that process. Xu et al. [52] observed elevated expression of miR-942-3p in tissue samples from HCC patients and cell lines. That was associated with disease and tumour node metastasis stages as well as shorter survival. The miRNA molecule was found to target the *MBL2* gene, and their levels were reciprocally related. Over-expression of MBL after transfection of LO2 HCC cells inhibited their viability and invasive potential. In contrast, MBL-knockdown in HepG2 cells had the opposite effect. In patients, low *MBL2* expression was associated with shorter survival, but no impact on tumour recurrence was noted. Based on multivariate regression analysis, *MBL2* gene expression level was concluded not to be an independent prognostic factor for overall survival (OS) [52]. However, earlier, Awan et al. [53] found that gene to be a target for a variety of deregulated microRNA molecules and considered it as a candidate HCC biomarker.

The expression of another collectin, CL-10, was found to be of prognostic value in HCC, with significantly lower *COLEC10* gene-specific mRNA and protein levels in abnormal compared with normal tissue samples. Low CL-10 expression was moreover associated with vascular invasion [54]. Later, Bai et al. [55] observed an elevated mRNA expression-based stemness index (mRNAsi) in HCC tissues and through weighted gene co-expression network analysis (WGCNA) identified *COLEC10* among 21 associated genes.

Low concentration of ficolin-2 was shown to confer a higher risk of liver cirrhosis and cancer in patients with chronic HBV infection [56]. Its level affects also the course of infection with HCV and effectiveness of anti-viral therapy [57]. It should be stressed that ficolin-2 is able to bind viral surface glycoproteins and inhibit pathogen entry to the host cells [58,59,60,61]. Chen et al. [56] and Yang et al. [61] reported decreased *FCN2* gene expression in HCC cells. Furthermore, it was shown that ficolin-2 (in association with transforming growth factor β, TGF-β) inhibits cancerous cell migration, epithelial–mesenchymal transition (EMT), and invasion. Patients with higher expression of this gene had lower risk of metastasis and longer disease-free survival [61].

Another member of the ficolin family, ficolin-3, was found to be deceased in HCC cells as well [30,62]. Moreover, proteomic analysis of plasma samples revealed lower levels of ficolin-3 in HCV-positive patients suffering from cancer than in those diagnosed with liver cirrhosis who did not develop HCC [62]. However, Ferrin et al. [63], with a larger group of patients, did not confirm that finding.

On the other hand, Jalal et al. [64] reported higher activity of mannose-binding lectin (as well as ficolin-2) in hepatitis C virus (HCV)-positive HCC patients, which may suggest that MBL concentration increases in response to chronic infection with that pathogen. Eurich et al. [65] postulated that carrying the X allele at position -221 of the *MBL2* gene is a risk factor for HCC associated with hepatitis C virus (HCV) infection in European subjects. Both X/X homozygosity and Y/X heterozygosity were associated with a larger tumour size, its bilobar growth, and higher pre-transplant α-fetoprotein concentration [65]. The relationship between this polymorphism and oncogenesis was confirmed in Chinese patients with hepatitis B virus (HBV) infection [66], those with and without HBV [51] and those without either HBV or HCV [67]. Furthermore, the B allele and YB haplotype (associated with low MBL concentration/activity) were found to be protective from developing liver cirrhosis and cancer [65], but the 54 A allele predicted better survival in cancer patients [66]. Interestingly, Su et al. [51] confirmed a beneficial effect of carrying the LYB haplotype but, as mentioned, considered the rare LXB variant as a risk factor for developing HCC.

In the case of pancreatic cancer, Rong et al. [68] suggested that MBL may be a disease marker, based on proteomic analysis of sera. Although in the case of pancreatic ductal adenocarcinoma, intraperitoneal bacteria expand markedly, from the perspective of this review, changes in composition of the fungal community seem to be most interesting; Aykut et al. [69] reported enrichment of the mycobiome infiltrating the tumour for *Malassezia* spp. and evidenced MBL-dependent complement activation upon recognition of surface structures to contribute to carcinogenesis. Moreover, using a murine model, they demonstrated that ablation of the mycobiome protects from cancer progression. Repopulation with *Malassezia* spp. (but not with *Candida* spp., *Saccharomyces* spp., or *Aspergillus* spp.) promotes oncogenesis [69].

Ytting et al. [70,71,72] reported significantly higher MBL concentrations in sera from Danish patients suffering from colorectal cancer in comparison with healthy controls. That finding was not reflected by differences in promoter or exon 1 *MBL2* allele distribution or by frequency of MBL deficiency. The lack of association of promoter/exon 1 polymorphisms (as well as two SNP localised to 3′UTR) with colorectal cancer was further confirmed by Lu et al. [73] in another Caucasian (Czech) population. In contrast, LYPA and LYQC haplotypes were found to be risk factors for CRC in African Americans [74]. Interestingly the first is associated with high but the second with lower MBL activity.

Proteomic analysis of sera demonstrated higher ficolin-3 expression in HCC patients infected with HBV in comparison with those without HBV or HCV infections [75]. Interestingly, Shen et al. [76] found significant changes of ficolin-3 concentration in sera from patients, after radiofrequency ablation. Those in whom it increased had better 1-, 2-, and 3-year disease-free survival rates than those experiencing a decrease. It should be however stressed that unexpectedly high ficolin-3 levels were reported (mean before and after treatment, 110.4 µg/mL and 289.7 µg/mL, respectively) [76]. In contrast, others have found the average serum concentration of ficolin-3 (not only in healthy individuals but also in cancer patients) not to exceed 40 μg/mL [28,77,78,79,80]. Recently, ficolin-3 was detected in ascites samples from patients with advanced pancreatic ductal adenocarcinoma (PDA) and peritoneal dissemination [81]. As mentioned above, dysbiosis may contribute to oncogenesis. In oesophageal squamous cell carcinoma and pre-malignant dysplasia, Verma et al. [82] found a higher expression of MASP-2 compared with normal cells. MASP-2 detected by immunohistochemistry employing a specific monoclonal antibody revealed its presence in both cytoplasm and nuclei of tumour cells. Furthermore, this high MASP-2 expression was correlated with advanced disease stage and metastasis [82]. The above-mentioned data confirm the multifaceted associations of LP factors with alimentary system cancers. Again, the site of their expression, disease type, stage, and the patient’s microbiome may be related to complement-dependent return to homeostasis or cancer progression.

## 5. Other Solid Tumours

Several reports concerned beneficial or harmful effects of LP-specific factors in cancers localized to other than reproductive, respiratory, or digestive systems. Jang et al. [30] reported lower *FCN3* gene expression in renal papillary cell carcinoma than in normal tissue. Interestingly, MBL was shown to inhibit ability of meprins (highly glycosylated zinc metalloproteases) to degrade components of extracellular matrix. Those enzymes are expressed in kidney and up-regulated in cancer cells [83]. Frederiksen et al. [79] observed lower concentrations of CL-10 and *MASP1* gene products (MASP-1, MASP-3, MAp44) in plasma from patients with head and neck cancer compared with healthy individuals. However, they found opposite relationships for ficolin-1 and ficolin-3 [79]. The concentrations of MBL and MASP-2 in serum were found to be higher in patients suffering from papillary thyroid carcinoma compared with healthy controls and patients diagnosed with thyroid adenoma [84]. Moreover, Lu et al. [73] reported higher *MBL2* gene expression in thyroid carcinoma cells compared with thyroid adenoma and normal cells. Fish et al. [85] found higher serum MASP-2 within, and serum MBL outside of the central nervous system (CNS) in paediatric patients diagnosed with solid tumours of the central nervous system (CNS). Furthermore, based on proteomic analysis of sera from patients and controls, ficolin-2 was proposed to be a candidate biomarker of oral cancer [86].

## 6. Concluding Remarks

**Figure 2:**
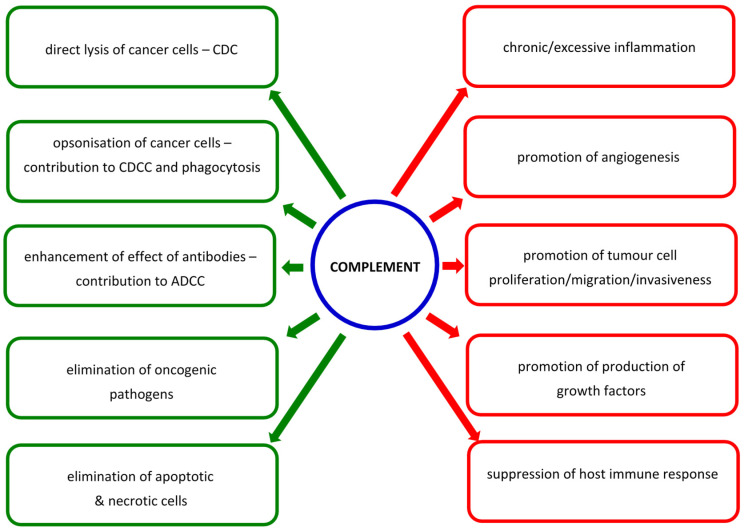
Multifaceted associations of complement with cancer. (**Left panel**)—effects beneficial for the host: complement activation leads to direct lysis of cancer cells (via formation of C5b9 complex; complement-dependent cytotoxicity, CDC), contributes to complement-dependent cellular cytotoxicity (CDCC) and phagocytosis (via opsonization of cancer cells with activated compounds), enhances the effect of antibodies (including therapeutic) (contribution to antibody-dependent cellular cytotoxicity, ADCC), and elimination of apoptotic/necrotic cells and some oncogenic pathogens. (**Right panel**)—effects detrimental for the host (mediated predominantly but not only by anaphylatoxins and/or formation of C5b9 complex): chronic inflammation may promote neoplastic transformation, uncontrolled complement activation may contribute to promotion of tumour cell proliferation/migration/invasiveness, angiogenesis, production of growth factors, and suppression of host response (especially via C5a action on myeloid-derived suppressor cells, which contributes to the decrease of CD8+ lymphocytes mediated by reactive nitrogen/oxygen species).

This review is focused strictly on factors specific for the lectin pathway of complement activation (pattern-recognizing molecules and associated serine proteases) but it has to be taken into consideration that LP activation leads to production of anaphylatoxins, formation of the membrane-attack complex, and interaction with other pathways, including the coagulation cascade. Complement-activating collectins and ficolins are characterized by relatively broad specificity, which allows for recognition of a variety of pathogen- or danger-associated molecular patterns (PAMP/DAMP). Their associated serine proteases contribute not only to the formation of C3 convertase but also (especially in the case of MASP-1) are able to cleave a variety of substrates, thus participating in cross-talk discussed in the introduction. As previously discussed [7,8,87,88,89,90,91], the complement system generally functions to prevent cancer through elimination of oncogenic pathogens or abnormal host cells, but complement activation may also promote the transformation of cells, their proliferation, tumour growth, epithelial–mesenchymal transformation, angiogenesis, or metastasis, and affect the outcome of immunotherapy (Figure 2). That reflects the high diversity of cancers and variety of mechanisms of complement action, especially in the tumour microenvironment and within cancer cells [87,88]. Therefore, complement-related therapeutic procedures have to be personalized and tailored. For example, Bareke and Akbuga [92] suggested that in treatment of ovarian cancer, a two-directional strategy sounds reasonable: promotion of its lytic activity but, on the other hand, inhibition of binding of anaphylatoxins to their receptors. Therapeutic modulation of complement activity appears a reasonable strategy in the treatment of at least some malignancies [7,93,94,95]. Components of the lectin pathway have also been considered as candidate biomarkers in certain cancers (Table 1), due to their altered concentrations in sera from patients and/or correlation with tumour grade/disease stage; however, that requires further investigation.

## Figures and Tables

**Table 1:** Components of the lectin pathway of complement as candidate biomarkers of solid tumour cancers.

Molecule	Disease (Association)	References
Mannose-binding lectin (MBL)	Pancreatic cancer (elevated protein expression; proteomic analysis of serum)	[68]
Collectin-10 (CL-10)	Hepatocellular carcinoma (decreased mRNA/protein expression)	[54]
	Colorectal cancer (low concentration in serum)	[96]
Collectin-11 (CL-11)	Lung adenocarcinoma (serum/urine concentration ratio)	[45]
Ficolin-1	Uterine corpus endometrial carcinoma (high *FCN1* gene expression)	[38]
	Colorectal cancer (high concentration in serum)	[96]
Ficolin-2	Uterine cervical cancer (high mRNA/protein expression)	[42,43]
	Oral cancer (elevated protein expression/proteomic analysis of serum)	[86]
Ficolin-3	Ovarian cancer (elevated expression/proteomic analysis of serum)	[41]
	Lung adenocarcinoma (decreased *FCN3* gene expression}	[30]
MASP-2	Colorectal cancer (high concentration in serum)	[71,97,98]
	Non-small lung cancer (high concentration in serum)	[47]
	Hepatocellular carcinoma (higher expression: transcriptome/secretome analysis; higher concentration in serum)	[99]
MAp44	Colorectal cancer (low concentration in serum)	[96]
